# Turing patterning with and without a global wave

**DOI:** 10.1371/journal.pbio.3000195

**Published:** 2019-03-25

**Authors:** Masafumi Inaba, Hans I-Chen Harn, Cheng-Ming Chuong

**Affiliations:** 1 Department of Pathology, Keck School of Medicine, University of Southern California, Los Angeles, California, United States of America; 2 International Research Center of Wound Repair and Regeneration (iWRR), National Cheng Kung University, Tainan, Taiwan; 3 Integrative Stem Cell Center, China Medical University Hospital, China Medical University, Taichung, Taiwan

## Abstract

Periodic patterning represents a fundamental process in tissue morphogenesis. In chicken dorsal skin, feather formation starts from the midline; then the morphogenetic wave propagates bilaterally, leaving a regular hexagonal array of feather germs. Yet, in vitro reconstitution showed feather germs appear simultaneously, leading to the hypothesis that the feather-forming wave results from the coupling of local Turing patterning processes with an unidentified global event. In this issue, Ho and colleagues showed such a global event in chicken feathers involves a spreading Ectodysplasin A (EDA) wave and Fibroblast Growth Factor 20 (FGF20)-cell aggregate-based mechanochemical coupling. In flightless birds, feather germs form periodically but without precise hexagonal patterning due to the lack of global wave.

## Abstract 2

Chicken feather buds form exquisite hexagonal patterns progressively from the midline to the flank, making them an excellent model of biological pattern formation. This Primer explores recent evidence that a local Turing periodic patterning, a global propagation mechanism, and mechano-chemical coupling work together to pattern the feather array progressively.

## Regular periodic feather patterns on the chicken skin

Animal integuments exhibit periodic patterns in spots, stripes, or mazes, which are made of pigment domains, hairs, or feathers. In the developing chicken embryo, one of the most fascinating phenomena is that feather buds begin to form at the dorsal midline after embryonic day 6, then propagate bilaterally toward the flank, leaving a highly ordered array of feather germs arranged in a hexagonal pattern after about three days [1–3].

**Fig 1:**
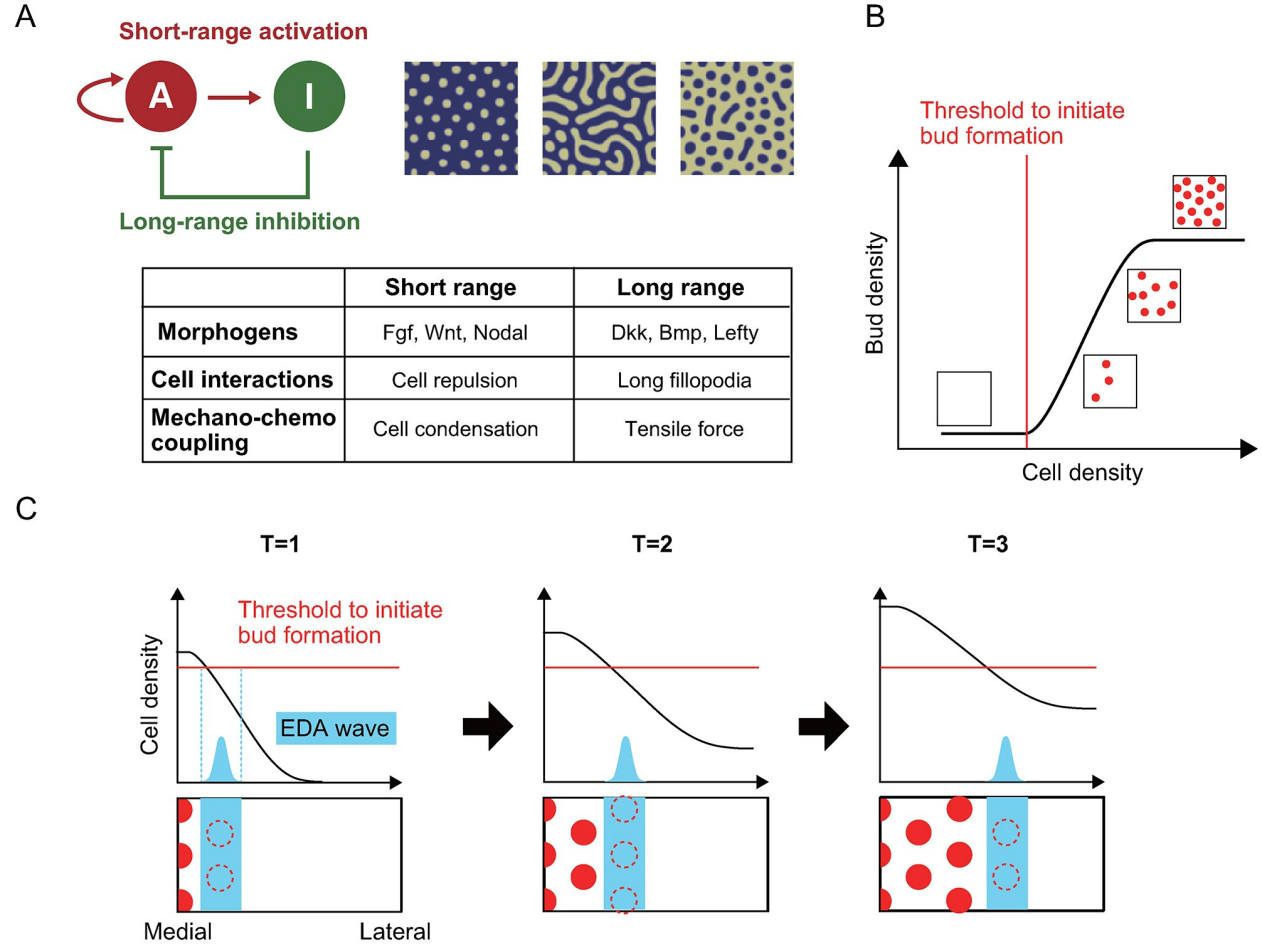
Turing model and its molecular cellular mechanisms. (A) Hypothetical molecular network generating Turing patterns. In the short range, an activator (“A”) enhances its own production and that of an inhibitor (“I”). In the long range, the inhibitor suppresses the production of the activator. Right figures are examples of resulting Turing patterns. Molecular factors and cellular interactions involved in the Turing model are summarized in the table. (B) Reconstitution of skin explants exhibit feather bud (red circles) formation depending on the mesenchymal cell density. The vertical red line represents the cell density threshold required to initiate bud formation. Toward the right, cell density reaches a level to form maximum bud density. (C) The horizontal red line represents the threshold required to form periodical feather primordia. Y axis reflects dermal cell density and the black curve represent dermal cell density schematically. A travelling EDA wave (blue) moving in the medial-lateral direction (x axis) at each time point (T1–3). EDA signaling adds to activator via mechanochemical coupling mechanism discussed in the text. Therefore, within the blue zone, less cell density is sufficient to generate periodically arranged feather buds. Red broken circles and red circles present feather buds during and after patterning, respectively. Panel B shows Turing patterning without global wave. Panel C shows Turing patterning with global wave, in this case, EDA wave. BMP, Bone Morphogenetic Protein; DKK, Dickkopf; EDA, Ectodysplasin A; FGF, Fibroblast Growth Factor.

Is this patterning process a playout of a molecular blueprint (like in *Drosophila*) or the result of stochastic local interactions? Perturbation experiments leading to altered patterns suggest the system is plastic [4,5], and progenitor cells for feather buds, including epidermal and dermal cells, are to form feather primordia. For local interactions, one fundamental theory is based on Turing’s reaction–diffusion model [6,7]. Turing showed hypothetical chemical reactions could form periodic structures spontaneously in a situation in which an activator activates its own production and a long-range inhibitor that represses the activator. When the inhibitor diffusion rate is much larger than that of activator, those chemicals are distributed heterogeneously, forming periodic patterns such as spots and stripes (Fig 1A).

In feathers, Fibroblast Growth Factor (FGF) and Bone Morphogenetic Protein (BMP) were shown to function as activators and inhibitors in Turing patterning [8]. Because the original Turing model assumes the initial condition is randomized, the resulting periodic patterns can vary stochastically. How does the regular hexagonal pattern happen? Experiments using reconstitution of a feather epithelium with dissociated mesenchymal cells help dissect the process and suggest that the propagation of the feather-forming wave results from the combination of a local Turing patterning process with a global signaling event [5]. In the in vitro experiment, the whole skin explant is a morphogenetic field. In vivo, with constraints imposed by the global event, a narrow stripe of the propagating morphogenetic field is the only place that can support periodic patterning that later spreads bilaterally, thus giving the orderly appearance. Mathematical modeling also predicts a traveling wave in tissue interactions during feather pattern formation [9]. However, the cellular and molecular basis of the global event and how the global event is coupled to the local Turing events were not identified previously.

Several more parameters must be considered when one thinks about the local interactions among skin progenitors. One being the intrinsic factors, which predetermines the responsive threshold of a cell, meaning cells must be in a competent state to respond to the activator and inhibitors in the environment. Molecularly, this can be translated as the number of morphogen receptors and the sensitivity threshold (e.g., FGF, BMP receptor, epigenetic states, etc.) or the amount of adhesion molecules expressed on the progenitors of feather buds. The other is the extrinsic factors, including the amounts of morphogens (e.g., WNT, FGF, BMP) or extracellular matrix molecules. These factors have to be within the right ratios and distribution to allow cells to launch Turing patterning.

Recently, the Turing concept has also been expanded beyond diffusible morphogens, and cell-cell adhesion or repulsion can mediate the activation or inhibitory function to reach Turing patterning without diffusion [10–12]. In the feather system, dynamic mesenchymal cell migration was observed [13], and local aggregation of mesenchymal cells and long-range tensile forces acting against tissue deformation may be caused by cell aggregation. Mesenchymal cell contraction may change β-catenin activity in epithelia and drive Wnt signaling, leading to the patterning [14]. It is the sum of these activators and inhibitors that drive the Turing patterning process within the morphogenetic field, whether they are in the form of diffusible morphogens or cell adhesive force, and whether they are generated by local or global events (Fig 1A).

On top of this local Turing event, when a directed global event breaks the symmetry, it can trigger Turing patterning on an asymmetric field [15], manifested as a propagating patterning wave. In this issue, Ho and colleagues [16] analyzed the molecular network that generates the periodic feather array and provide new clues for us to understand the molecular circuit operating in the propagation of the morphogenetic wave.

## FGF20/ BMP4 feedback loop that facilitates Turing patterning locally

Ho and colleagues began by investigating the relationship between the FGF20/BMP4 pathway and mesenchymal cell aggregation in developing chick feathers. When beads soaked with FGF9 protein are placed on the competent skin field, mesenchymal cell aggregation and FGF20/BMP4 up-regulation were observed. This up-regulation was inhibited if mesenchymal cell condensation was suppressed by an inhibitor for cell migration. This means cell aggregation plays a critical role in inducing downstream signals. Furthermore, BMP signals inhibit FGF expression. Therefore, FGF signaling forms a localized positive feedback loop through mesenchymal cell aggregation, and the BMP signal works as a long-range inhibitor of FGF expression. This network has the basic characteristics required for Turing reaction–diffusion production of periodic patterning.

## Ectodysplasin A wave as the global event

Based on the observed order of feather appearance in chicken embryos, the authors asked how the regular periodic pattern is formed. A computer simulation based on their findings suggests the existence of a traveling wave interacting with the Turing model factors could produce the highly ordered hexagonal feather array and sequential feather formation from the midline to the lateral edge of the skin. When cell migration is suppressed transiently ex vivo, the order of feather array was distorted, suggesting the traveling wave is important in organizing molecular signaling and cell aggregation for feather patterning.

What is the cellular and molecular bases of this traveling wave? Recent studies imply that mechanical properties may play a role in feather array formation [14]. To evaluate whether mechanical force is the basis of the global wave, Ho and colleagues cut a piece of the skin explant and kept it away from the scaffold to allow the explant to contract in in vitro culture. Local tissue contraction did not change the position and the timing of feather formation, implying mechanical force is not the global wave and different factors are required to guide the propagation of the feather forming wave.

The mRNA expression of Ectodysplasin A (EDA) suggests it may be a candidate [17]. It first emerges in the midline as a longitudinal stripe, then spreads bilaterally. EDA is a diffusible protein and is shown to induce FGF20 expression through binding of the EDA receptor (EDAR). In this process, beta-catenin (CTNNB1) was observed to be expressed globally in the dorsal skin, defining the morphogenetic field [5]. As the EDA wave progresses laterally, the global CTNNB1 expression regresses and is replaced by enhanced CTNNB1 expression in each feather primordium that forms from the midline to the lateral edge. This moving wave front helps define the precise position of newly formed feather primordia.

When the EDA pathway is up-regulated, width of FGF20 expression region is increased. Conversely, down-regulation of the EDA pathway decreased the width of the FGF20 expression zone. In FGF20 chicken mutant skin, the EDA wave is still observed. Therefore, FGF20 signaling is not required for global wave propagation.

Cell density has been shown to set up the threshold of feather formation (Fig 1B). Using the skin reconstitution experiments with different mesenchymal cell density, feather germs start to emerge when the cell density reaches a threshold to launch Turing patterning. But the hexagonal-like feather array is not reached until feather germs reach the highest packing density [5]. Ho and colleagues further investigated the interaction between EDA waves and mesenchymal cell density (Fig 1C). Reducing cell density by inhibiting cell proliferation led to a narrower feather tract—avian skin regions where feathers can grow—yet the EDA expression wave was not affected. Therefore, mesenchymal cell density does not control the molecular wave. Yet, EDA seemingly affects the mesenchymal cell property that senses the environment. Activation of EDA appears to allow mesenchymal cells to initiate periodic patterning at lower cell density through FGF20 induction. Therefore, mesenchymal cells can sense their environment (cell density in this case) but their function (periodic patterning) is regulated by the chemical factor, EDA.

## Cell adhesion and a mechanochemical coupling loop

Mechanical force has now been shown to be one of the driving forces in development. Cell growth, as it increases cell density, generates mechanical stress in the environment surrounding the growing cell. These mechanical interactions are shown to be essential for many morphogenetic processes as seen in *Drosophila* development [18] and limb skeletal morphogenesis [19]. Feather primordium formation is characterized by dermal condensation and epidermal placode formation. Dermal condensation is a process of increased local dermal cell density caused by increased cells migration and adhesion. When Latrunculin A, an inhibitor of actin filament polymerization, abrogated cell migration by hampering force generation, it in turn disrupted primordium formation. In feathers, Neural Cell Adhesion Molecule (N-CAM) serves as one of the adhesion molecules mediating dermal condensation formation [5].

Directional cell migration of both epithelial and dermal cells also plays a key driving force behind hair placode morphogenesis during mouse skin development [20,21]. Externally applied force via cell constraint activates Pax9 in a mesenchymal condensation during embryonic tooth germ formation [22]. Together these findings show that mechanical force, achieved through high cell density, cell migration and/or adhesion, could serve as an activator that turns on key signals required to engage cell collectives in Turing patterning. We can also state that mechanical force contributes to the side of Turing activator, and it is the sum of Turing activator and inhibitors that cells use to make decisions on whether to enter Turing patterning (Fig 1A). Therefore, this is a chemomechanical coupling event that should be fundamentally observed in many other model systems.

## Irregular feather patterns on the skin of flightless birds: Emus and ostriches

**Fig 2:**
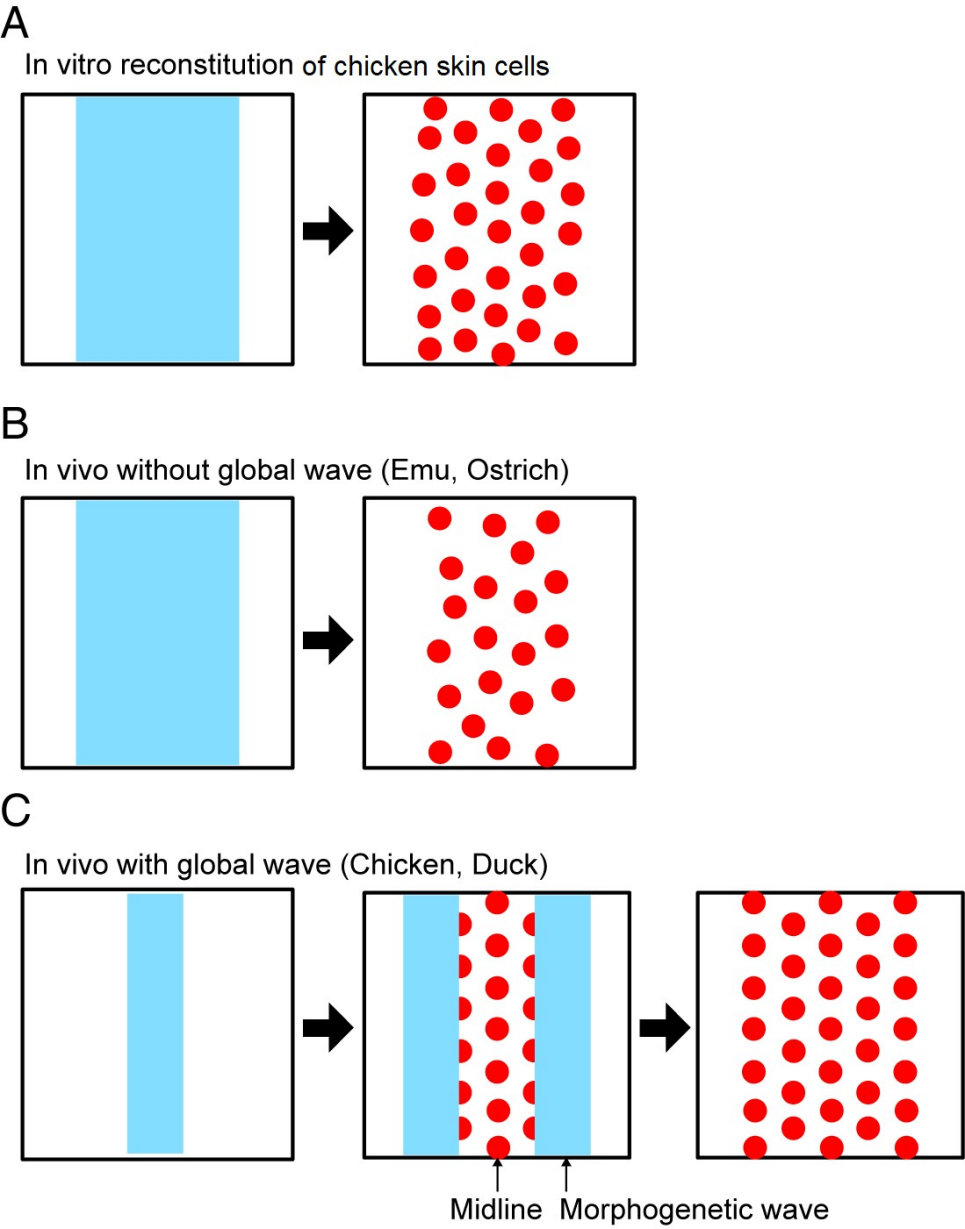
Feather array formation with and without a global wave. (A) In the in vitro reconstituting chicken skin explant, the morphogenetic field (blue) is experimentally static, leading to the simultaneous formation of a less ordered feather (red circle) array. (B) In flightless birds, this less ordered feather array is formed because it is based only on the Turing patterning principles in a local static morphogenetic field. (C) In in vivo flight birds, the high ordered feather array is formed sequentially from the midline to lateral regions by the morphogenetic waves that travel bilaterally.

Ho and colleagues then compared the distribution of feather buds on the trunk of flightless birds. Feather buds on ostrich and emu embryos tend to show less ordered feather arrays as compared with those in flying birds. Further analyses showed ostrich embryos display no EDA wave, which seems to be the reason for less ordered feather patterns. In emus, the mechanism appears to be different. Emu skin does not exhibit a global EDA wave either. It shows molecularly defined feather tract regions in embryonic development that do not produce feathers until later. In emus, the densification of mesenchymal cells is extremely delayed, missing the timing to interact with molecular factors. Therefore, ostriches and emus may have independently acquired different ways to keep their irregular feather arrays. This irregular periodic patterning may be due to the lower demand to acquire regularly arranged contour feathers required for flight. Therefore, Turing patterning can form in combination or not in combination with a global traveling wave to generate different feather array patterns (Fig 2).

## Outlook

The periodic feather arrays are formed by local cell–cell interactions that satisfy the requirements needed for Turing patterning. This system can define the periodicity of the pattern but cannot set the specific feather array pattern. Global mechanisms such as traveling waves that traverse the whole skin generate the timing and positioning of patterning within a morphogenetic field.

Biological waves act at many levels in living systems: calcium ion waves after frog egg fertilization [23], actin assembly waves in cell migration [24], cyclic Adenosine Monophosphate (cAMP) waves leading to slime mold pattern formation [25], organ differentiation in *Drosophila* eyes [26], and cyclic regeneration of hair follicle population [27], etc. Given that our body is composed of highly ordered tissues, a combination of local and global control may be a fundamental process to reinforce the accuracy and robustness of morphogenesis.

This work has nicely presented the molecular components of the global wave in feather array formation and how the global wave is coupled to the local Turing patterning process. They also elegantly use emu and ostrich skin to contrast the different patterning mechanisms. Yet there are also many unsolved questions. For example, the authors did not explore how the EDA wave propagates. How EDA modulates the threshold of mesenchymal cells in the context of mechano-chemical coupling will need to be elaborated in future studies. How do mesenchymal cells sense the environment? When will the EDA wave stop and how the tract boundaries are set are also interesting unsolved questions. This paper is a good step toward understanding these wonderful biological examples of periodic pattern formation.

## Abbreviations

BMP: Bone Morphogenetic Protein
cAMP: cyclic Adenosine Monophosphate
CTNNB1: Catenin Beta 1
EDA: Ectodysplasin A
EDAR: EDA Receptor
FGF: Fibroblast Growth Factor
N-CAM: Neural Cell Adhesion Molecule
DKK: Dickkopf
